# High-resolution imaging and three-dimensional model of the feline spinal cord

**DOI:** 10.3389/fnana.2026.1764931

**Published:** 2026-02-18

**Authors:** Jonathan Harnie, Oussama Eddaoui, Khaled Ashkar, Charlène Nadeau, Stephen Mari, Sirine Yassine, Rasha Al Arab, Johannie Audet, Christian Iorio-Morin, Alain Frigon

**Affiliations:** 1 Department of Pharmacology-Physiology, Faculty of Medicine and Health Sciences, Centre de Recherche du CHUS, Université de Sherbrooke, Sherbrooke, QC, Canada; 2 Division of Neurosurgery, Centre de Recherche du CHUS, Université de Sherbrooke, Sherbrooke, QC, Canada

**Keywords:** feline model, magnetic resonance imaging, micro-computed tomography, spinal cord, three-dimensional model, finite element analysis

## Abstract

The spinal cord is a critical component of the central nervous system, responsible for integrating somatosensory inputs, generating motor outputs, and regulating autonomic functions. Despite its functional importance, high-resolution anatomical data spanning from high cervical to sacral levels of the spinal cord remain largely unexplored, limiting our ability to develop accurate surgical strategies, computational models, and neuromodulation protocols. Here, we performed a segment-by-segment quantitative anatomical analysis of the feline spinal cord from C3 to S2 using histology and high-resolution micro–computed tomography (Micro-CT) to evaluate key structures including the dura mater, dorsal root ganglia, rootlets, and white and gray matter. We observed significant variability in dural thickness, as well as structured changes in rootlet orientation and density along the spinal axis. Dorsal root ganglia size, along with white and gray matter morphology, varied across spinal segments, with prominent enlargements at cervical and lumbar levels. Additionally, we constructed an open-access three-dimensional model by integrating magnetic resonance imaging (MRI), Micro-CT, and high-resolution Micro-CT data into a unified spatial reference. This model enables precise spatial analysis of spinal structures and facilitates advanced computational modeling of spinal cord function and neuromodulation strategies. Our results represent a valuable resource for anatomical, surgical, and bioengineering applications aimed at improving spinal interventions.

## 1 Introduction

The spinal cord plays an essential role in the central nervous system for controlling movement, processing somatosensory information and regulating various autonomic functions. Advancing our anatomical understanding of the spinal cord’s structure is key for identifying factors to improve surgical interventions and neuromodulation approaches, such as electrical spinal cord stimulation, which has been used to reduce chronic pain (Lempka and Patil, 2018) or facilitate motor recovery in humans (Angeli et al., 2018; Gill et al., 2018; Wagner et al., 2018). Despite its importance, there is a notable lack of high-resolution anatomical data describing the spinal cord across its full rostrocaudal extent.

Traditionally, anatomical characteristics of the spinal cord have been studied through dissection and histological techniques (Mendez et al., 2021; Pearce, 2008). However, these invasive techniques often deform or remove essential tissues or components, such as vertebrae, dura mater and/or cerebrospinal fluid. Improvements in medical imaging have greatly advanced our anatomical understanding of the spinal cord by eliminating the need for direct tissue manipulation while providing high-resolution isotropic 3D visualization of both soft and hard tissues through magnetic resonance imaging (MRI) and X-ray computed tomography (CT), respectively (Florkow et al., 2022; Ruiz Santiago et al., 2022). However, both techniques have limitations. For example, while MRI offers excellent contrast resolution, the spatial resolution in typical clinical MRI scanners is generally limited to about 0.2–1.0 mm in-plane, constrained by scan time and diminishing contrast-to-noise returns as voxel size decreases. This resolution is insufficient to properly visualize very small structures like rootlets, which are often only a few hundred micrometers in diameter, although a recent study using diffusion-based tractography demonstrated the feasibility of inferring the organization of these structures *in situ* (Liang et al., 2025). Conversely, CT scans, which are ideal for imaging bones, are less effective at visualizing soft tissues. Micro-computed tomography (μCT) has recently gained popularity as a method for obtaining high-resolution images of neural structures in rats (Farrag et al., 2021; Jiang et al., 2020), mice (Girard et al., 2016; Park et al., 2014), and pigs (Chin et al., 2024).

The cat model has been and remains an important model for studying spinal cord anatomy and its circuits (Jankowska et al., 1967; Sherrington, 1910; Yakovenko et al., 2002) as well as the control of autonomic (Tai et al., 2008; Wang et al., 2021) and sensorimotor functions, such as locomotion (Frigon et al., 2021; Grillner and Zangger, 1979; Rossignol and Frigon, 2011). However, no studies have characterized the anatomical morphology of the feline spinal cord using high-resolution imaging, which shares many characteristics with the human spinal cord (Toossi et al., 2021). Although the lumbar and sacral levels are primarily targeted due to their critical role in lower limb control, conducting a comprehensive analysis across the entire spinal cord remains essential for other functions, such as forelimb control and autonomic functions.

Therefore, the goal of this paper was first to provide a quantitative anatomical analysis of the feline spinal cord from C3 to S2, using histology and high-resolution Micro-CT imaging to assess key structures, including the dura mater, dorsal root ganglia (DRG), rootlets, white matter, and gray matter. Secondly, by integrating MRI, Micro-CT and high-resolution Micro-CT imaging, we provide a comprehensive open-access 3D model that can serve as a valuable resource for anatomical, computational, and neuromodulation research.

## 2 Materials and methods

### 2.1 Animals and ethical information

All procedures were approved by the Animal Care Committee of the Université de Sherbrooke and were used in accordance with policies and directives of the Canadian Council on Animal Care (Protocol 2022–3,349). Four adult domestic short-haired cats, 2 males and 2 females, aged 317, 325, 435, and 793 days and weighing between 3.35 and 5.1 kg (4.37 ± 0.81 kg), were used in the present study. We followed ARRIVE guidelines for animal studies (du Percie Sert et al., 2020). Before and after experiments, cats were housed and fed in a dedicated room within the animal care facility of the Faculty of Medicine and Health Sciences at the Université de Sherbrooke. All cats were used in other studies to maximize their scientific output.

### 2.2 General experimental protocol

**Figure 1:**
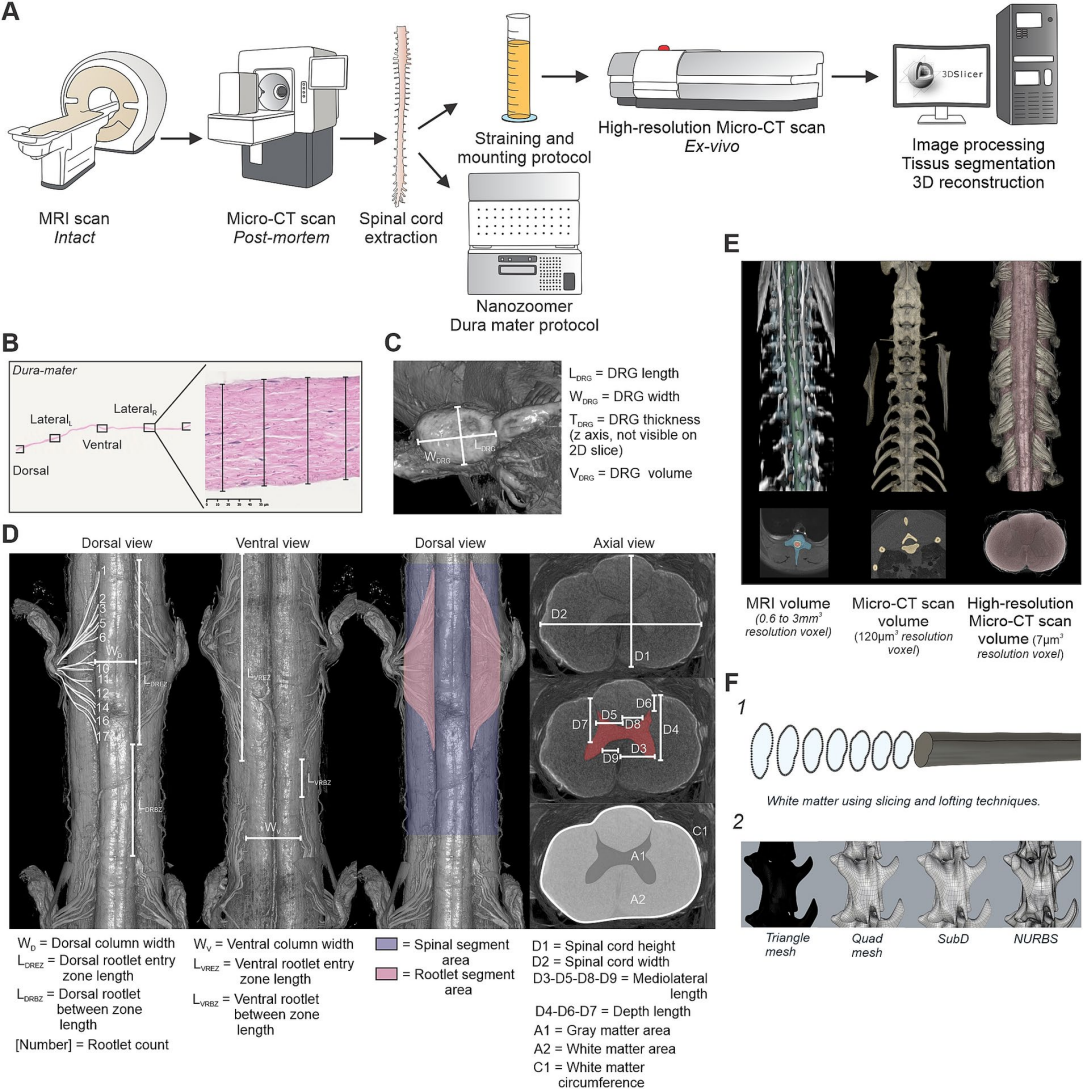
General methodology for multimodal acquisition, analysis, and modeling of the feline spinal cord. **(A)** Overview of the experimental pipeline. MRI scans were first acquired on the intact animal, followed by post-mortem Micro-CT imaging (MicroCTpm). The spinal cord was then extracted for histological processing (dura mater) and high-resolution *ex vivo* Micro-CT scanning (MicroCTex). Images were segmented and processed in 3D Slicer to enable tissue labeling and 3D reconstruction. **(B)** Dura mater analysis. Four measurements were performed on histological slices (H&E stained) at predefined anatomical locations: dorsal, ventral, and both lateral sides. **(C)** Dorsal root ganglia (DRG) morphometric analysis. Length (L_DRG_), width (W_DRG_), thickness (T_DRG_), and volume (V_DRG_) were extracted from MicroCTex images. **(D)** Rootlet and spinal cord morphometry. Dorsal and ventral views were used to quantify rootlet width, entry zone lengths, and number of rootlets. Rootlet segment area and spinal segment area were quantified along the cord. In axial view, white matter, gray matter, and global spinal dimensions (D1–D9, A1, A2, C1) were extracted. **(E)** Example volumes obtained from the three imaging modalities: MRI, MicroCTpm, and MicroCTex. Segmentations were performed and aligned to the MicroCTpm space. **(F)** 3D model simplification. **(F**_**1**_**)** Lofting method using 200 axial white matter profiles to generate simplified solid models. **(F**_**2**_**)** Conversion of triangle meshes to quad meshes, SubD, and NURBS surfaces for vertebrae.

The general protocol of this study is illustrated in Figure 1A. We first performed MRI scans (Ingenia 3.0 T, Philips Healthcare, Best, The Netherlands). After being used for other experimental objectives, the animal was euthanized with a lethal dose (120 mg/kg) of pentobarbital via the left or right cephalic vein. We then conducted postmortem Micro-CT imaging (MiLabs, Utrecht, The Netherlands) before extracting the spinal cord from the vertebral column. The extracted tissue was then processed following dedicated protocols, with the dura matter prepared for histological analysis and the spinal cord treated to enhance imaging quality (see below). Finally, high-resolution *ex-vivo* Micro-CT scans (Skyscan 1,172, Bruker Micro-CT, Kontich, Belgium) were conducted on the extracted spinal cord followed by image processing, segmentation, and 3D reconstruction.

### 2.3 Spinal cord extraction

To extract the spinal cord, we placed the animal in a prone position. The skin overlying the dorsal aspect of the vertebral column was excised along the length of the spine using surgical scissors. Next, incisions were made alongside the spinous processes, laminae, and transverse processes to expose the vertebrae’s spinous processes. Using sharp prong retractors or a scalpel, we retracted the paraspinal muscles to expose the posterior elements of the spine. Using rongeurs, we performed a dorsal laminectomy to expose the entire spinal cord. Then, we carefully lifted the spinal cord from the caudal end and severed the spinal nerves just distal to the DRG. The approximately 40 cm of extracted spinal cord was cut into two equal segments and placed in 500 mL tubes containing 4% paraformaldehyde solution in a 0.1 m phosphate buffer solution (PBS) at 4 °C. After 5 days, the spinal cord was cryoprotected in PBS with 30% sucrose for 72 h at 4 °C.

### 2.4 Histology

Before preparing the spinal cord for high-resolution *ex-vivo* Micro-CT imaging, the dura mater was carefully removed circumferentially between each nerve root using microsurgical instruments. Once dissected, each section was individually placed in a cassette, immersed twice for 5 min in 70% industrial methylated spirits (IMS), and then soaked for 24 h in fresh 70% IMS. The tissues were then completely dehydrated using the following protocol: 1 h each in 70, 75, 90, and 95% IMS, followed by three immersions in 100% IMS for 1 h, 1 h 30 min, and 1 h 30 min, respectively. They were subsequently cleared in three 1-h baths of 100% xylene and finally infiltrated with paraffin wax during two incubations of 2 h 20 min each. We mounted 4 μm slices on glass slides for each spinal segment and then stained them using hematoxylin and eosin following a standardized protocol (Lee et al., 2002). High-resolution imaging of each slide was performed using a NanoZoomer microscope (Hamamatsu Photonics, Hamamatsu, Japan) for detailed visualization and quantitative morphological analysis.

### 2.5 Spinal cord imaging

#### 2.5.1 MRI

We performed T2 FatSat axial (TR, 4,400 ms; TE, 80 ms; FOV, 70 mm; section thickness, 3 mm; 50 sections without a section gap; matrix size, 224 × 224; in-plane resolution, 0.2232 × 0.2232 mm^2^; acquisition time, 15 min and 24 s), T2 sagittal (TR, 3,000 ms; TE, 80 ms; FOV, 170 mm; section thickness, 3 mm; 13 sections without a section gap; matrix size, 1,008 × 1,008; in-plane resolution, 0.2282 × 0.2282 mm^2^; acquisition time, 15 min and 5 s) and 3D T2 SPAIR lumbar cord images (TR, 1,300 ms; TE, 276 ms; FOV, 230 mm; section thickness, 0.6 mm; 166 sections without a section gap; matrix size, 800 × 800; in-plane resolution, 0.2875 × 0.2875 mm^2^; acquisition time, 15 min and 15 s) using a 3 T magnetic resonance scanner (Philips Healthcare, Best, The Netherlands) equipped with a Philips dStream small extremity coil. During acquisition, the cat was under anesthesia and in a supine position. Cats were sedated with an intramuscular injection containing ketamine (2 mg/kg), butorphanol (0.2 mg/kg), dexmedetomidine (0.2 mg/kg). Cats were then anesthetized with isoflurane (1–3%) delivered in O_2_, first with a mask and then with an endotracheal tube. During image acquisition, we adjusted isoflurane concentration by monitoring cardiac and respiratory rates. Earplugs and earmuffs were placed in and over the animal’s ears to protect it from the machine’s noise.

#### 2.5.2 Post-mortem Micro-CT scan (MicroCTpm)

After euthanasia, we collected images using a Micro-CT scanner (MiLabs, Utrecht, The Netherlands). The cat was placed in a prone position. The images were reconstructed with a resolution of 120 μm × 120 μm × 120 μm, tube voltage of 55 kV, tube current of 0.33 mA, an exposure time of 40 ms, and a 0.125 degree step angle.

#### 2.5.3 *Ex-vivo* high-resolution Micro-CT scan (MicroCTex)

To achieve higher resolution, after spinal cord extraction, we performed *ex-vivo* imaging using a high-resolution Micro-CT scanner (Skyscan 1,172, Bruker Micro-CT, Kontich, Belgium). The tissue was prepared following the procedure described by Strotton et al. (2018). After extraction and treatment in paraformaldehyde and sucrose solutions, we removed the dura mater, and a 25% Lugol’s Iodine (LI) solution was made by diluting a 100% LI stock solution (10 g KI and 5 g I₂ in 100 mL H₂O). The tissues were stained in 25% LI for 48 h, followed by removal of excess stain through three 24-h PBS washes. The tissues were then dehydrated in IMS with 8-h incubations at 30, 50, and 70%. Complete dehydration was achieved with 2-h washes in 90% IMS and three washes in 100% IMS, followed by clearing in Xylene-IMS (1:1 for 2 h and three rounds of Xylene) and paraffin wax infiltration (two 2-h rounds in paraffin wax). Before drying, the spinal cord was sectioned into segments approximately 6 cm long. Images were reconstructed at a resolution of 7 μm × 7 μm × 7 μm, with a tube voltage of 40 kV, tube current of 250 μA, exposure time of 450 ms, and a step angle of 0.4 degrees.

### 2.6 Data analysis

#### 2.6.1 Dura mater

The dura mater was analyzed using histological techniques to assess its thickness across different spinal segments. Thickness measurements were taken at four predefined locations: dorsal, ventral, lateral left, and lateral right, using NDP.view2 software (Hamamatsu Photonics, Hamamatsu City, Japan) (Figure 1B). Due to the natural variability in dura mater thickness within the same sample, four measurements were taken for each side (dorsal, ventral, lateral left, and lateral right), and the mean value was calculated to ensure accuracy and reproducibility. Data were collected for the group (*n* = 4 cats, C3-L7). Based on the final 3D model from one cat, we measured the dural circumference at each spinal segment (C3-S2).

#### 2.6.2 Dorsal root ganglia

The data on the DRG were analyzed from images acquired using MicroCTex (Figure 1C). DRG length (L_DRG_), width (W_DRG_), thickness (T_DRG_) and volume (V_DRG_) were quantified across spinal levels. In some cases, a portion of the DRG was missing, however, a complete analysis was successfully performed on at least one DRG per spinal level, except for one cat at C8 and T1, where measurements could not be obtained. Consequently, DRG data were analyzed and reported for three cats (C3-S2).

#### 2.6.3 Rootlet, white, and gray mater

To quantitatively assess spinal cord and rootlet morphology, we measured several parameters (Chin et al., 2024; Mendez et al., 2021; Toossi et al., 2021), based on MicroCTex imaging (Figure 1D). The dorsal view was used to quantify the dorsal column width (W_D_), the dorsal rootlet entry zone length (L_DREZ_), and the dorsal rootlet between-zone length (L_DRBZ_). Similarly, the ventral view allowed for the measurement of ventral column width (W_V_), the ventral rootlet entry zone length (L_VREZ_), and the ventral rootlet between-zone length (L_VRBZ_). Data were collected for the group (*n* = 4 cats, C3-S2). Furthermore, the proportion of rootlets relative to the white matter was quantified in dorsal, ventral, and lateral views. To determine this proportion at each spinal segment, the segment area (blue region) and the rootlet area (pink region) were extracted and analyzed. Data were collected for the group (*n* = 4 cats, C3-S2). We also quantified the number of rootlets in one cat (Table 1). Finally, cross-sectional morphometric parameters of the spinal cord were analyzed in axial views. Measurements included spinal cord height (D1) and width (D2), mediolateral distance from the midline to the lateral boundary of the ventral horn (D3), depth from the dorsal surface of the spinal cord to the ventral boundary of the ventral horn (D4), mediolateral distance from the midline to the lateral boundary of the dorsal horn (D5), depth from the dorsal surface to the dorsal boundary of the dorsal horn (D6), depth from the dorsal surface to the dorsal boundary of the ventral horn (D7), mediolateral distance from the midline to the medial boundary of the dorsal horn (D8), mediolateral distance from the midline to the medial boundary of the ventral horn (D9), cross-sectional areas of the white matter (A1) and gray matter (A2) and white matter circumference (C1) at each spinal level (Figure 1D, axial view). Data were collected for the group (*n* = 4 cats, C3-S2).

**Table 1:** Number of rootlets in one cat.

Side	C3	C4	C5	C6	C7	C8	T1	T2	T3	T4	T5	T6	T7	T8
Dorsal	28	21	24	31	37	31	19	10	11	9	8	8	11	12
Ventral	19	21	25	52	55	59	32	17	11	18	20	14	20	16

Side	T9	T10	T11	T12	T13	L1	L2	L3	L4	L5	L6	L7	S1	S2
Dorsal	12	6	10	11	10	12	11	13	19	14	20	32	17	16
Ventral	16	11	20	18	15	22	18	12	17	41	54	35	27	14

The table shows the number of rootlets at each spinal segment from the third cervical segment (C3) to the second sacral segment (S2). Counts were performed separately for the dorsal and ventral sides.

#### 2.6.4 Cerebrospinal fluid

To characterize the morphological properties of the cerebrospinal fluid (CSF), we measured volume and thickness on a rendered 3D model (see below). These data were obtained from a single cat (C3-S2). The volume was calculated at each spinal segment and summed to obtain the total volume. The thickness was measured at all four anatomical positions (dorsal, ventral, lateral left, and lateral right).

### 2.7 Image processing

MRI and Micro-CT datasets registration was performed using a combination of two open-source software packages: Spinal Cord Toolbox (SCT) (De Leener et al., 2017), and 3D Slicer (Fedorov et al., 2012). Among the MRI sequences acquired, only the T2 FatSat axial sequence was used for registration due to its high contrast for deep spinal cord tissues. An initial registration step involved spinal cord straightening to facilitate alignment across modalities. For MRI data, the *sct_straighten_spinalcord* program from SCT was employed, while for CT data, the *Curved Planar Reformat* module from 3D Slicer was used. MicroCTpm was designated as the fixed reference volume for all subsequent registration procedures. Linear alignment was then performed on MRI data and MicroCTex to align them with the fixed reference volume, utilizing the *Transform* module in 3D Slicer. Due to the loss of spinal cord tension following ex vivo dissection and tissue preparation, anisotropic scaling was applied to MicroCTex data to restore tissue volume to its original *in vivo* dimensions. Specifically, scaling factors were determined based on measurements from the intact spinal cord MRI data, resulting in an average scaling of 45% in the longitudinal (z) axis—compensating for elongation due to the release of tension during dissection—and 10% in the transverse (x and y) axes to account for volumetric reduction from dehydration during tissue processing. Finally, the *Landmark Registration* module in 3D Slicer was used to apply a thin-plate spline (TPS) deformation to both the MRI and MicroCTex volumes to achieve precise alignment and centering of the spinal cord within the spinal canal of the MicroCTpm scans. Although TPS is a nonlinear transformation, in this case it was applied in a controlled manner—primarily to refine alignment within individual axial planes. Multiple fiducial landmarks were placed per axial slice and adjusted collectively to ensure consistent in-plane (x/y) positioning with minimal deformation. Because the volumes had been pre-straightened, this approach introduced only minor bending necessary to ensure the spinal cord conformed accurately to the canal geometry.

### 2.8 3D model generation

The MRI, MicroCTpm and MicroCTex acquisitions were used to generate a unified anatomical 3D model of the cat’s spine. The various tissues were segmented using 3D slicer (Figure 1E). The vertebrae were segmented on the MicroCTpm directly in the reference space. The experimental protocol described in the present study is part of a broader project dedicated to three-dimensional motor mapping of the lumbar spinal cord. As a consequence, the lumbar vertebrae included in the 3D model underwent a dorsal laminectomy to allow direct access to spinal circuits during functional mapping experiments. The comprehensive motor mapping study is currently under preparation for publication. Dura mater and CSF were also segmented in the reference space but were based on coregistration of the MicroCTex and the axial MRI T2 FatSat sequence superimposed in the background. This approach ensured resolution consistency across all tissue segmentations inside the spinal canal. The spinal cord’s gray matter, white matter, and rootlets were segmented directly on the MicroCTex in its native acquisition space and transferred to the reference space using the *Transform* module in 3D Slicer, applying the saved transformation matrices from the image processing step.

All segmentations were then converted into 3D meshes using 3D slicer and exported in. STL file format. Due to high resolution of CT data, segmentations spanning the entire spinal cord (C3 to caudal end), including gray and white matter, CSF, and vertebrae, produced 3D meshes too large for practical applications. To address this, the following steps were implemented to simplify and convert meshes into solid models:

#### 2.8.1 Simplification of gray and white matter, cerebrospinal fluid, and dura mater

A custom Python script was used to extract 200 evenly spaced axial profiles along the rostro-caudal axis of the spinal cord. The script is publicly available in the Zenodo repository associated with this study. Each profile consisted of a spline curve with 40 points fitted to the cross-sectional geometry of the mesh. These profiles were imported into Fusion 360 (Autodesk Inc., San Rafael, CA, United States), where the *Loft* operation was applied to generate solid models from the interpolated cross-sections (Figure 1F_1_).

#### 2.8.2 Simplification of vertebrae and rootlets

Given the complex geometry of the vertebrae, direct lofting was not feasible. Instead, the triangular meshes were processed in Rhino 8 (Robert McNeel and Associates, Seattle, WA, United States) by (1) converting the triangle mesh into a quad mesh; (2) refining into subdivision surfaces (SubD); and (3) converting the resulting SubD models into non-uniform rational B-splines (NURBS) (Figure 1F_2_). The same simplification can be applied to the rootlets. However, because the simplified rootlet models could not be properly utilized in COMSOL, these data were not included for sharing. The resulting solid geometries were exported in. STL and. STEP formats for further use. The data set are openly available on Zenodo under a CC-BY 4.0 license: 10.5281/zenodo.15333382.

### 2.9 Finite element analysis software compatibility

The computer used for model generation and testing was equipped with an Intel Core i7-10700 CPU (2.9 GHz), NVDIA RTX 3080, 128 GB of RAM, and was running Windows 10. The compatibility and operational use of the generated models were tested using two software platforms: Sim4Life (v7.2.4, ZMT Zurich MedTech AG, Zurich, Switzerland) and COMSOL Multiphysics (v5.5, COMSOL AB, Stockholm, Sweden). For both platforms, testing was conducted using only the internal geometry handling and meshing tools provided by each software.

#### 2.9.1 Sim4Life

The full model, including gray and white matter, cerebrospinal fluid, dura mater, vertebrae, and rootlets/roots, was imported for testing. A low-frequency ohmic quasi-static simulation (a basic placeholder simulation) was used to evaluate the voxelization of the model. Materials and boundaries were left as default, and automatic “very fine” grid settings were applied to all components except the dura. For the dura, a custom grid size of 0.06 × 0.06 × 0.1 mm was empirically determined to ensure accurate voxelization of this thin structure. Voxel priority was assigned hierarchically, starting with the internal structures (gray matter having the highest priority), followed by white matter, rootlets/roots, cerebrospinal fluid, dura mater, and vertebrae. This priority system resolved intersections between domains during voxelization.

#### 2.9.2 COMSOL Multiphysics

In COMSOL, testing was conducted using the model without rootlets, as their complex geometry introduced major unresolved errors during the final union operation within COMSOL’s geometry node. All intersecting domains were separated using the *Boolean difference* operation to maintain proper geometry representation. Meshing was set to the preset “extremely fine” for all structures except for the vertebrae, where a minimum element length of 0.00008 mm was used to accommodate their intricate details.

### 2.10 Statistical analysis

All statistical analyses were performed using Friedman’s test to evaluate the effect of spinal segment and anatomical location on measured parameters. Specifically, we applied Friedman’s test to assess the impact of spinal segment variations on dura mater thickness, morphological DRG dimensions and volume, rootlet dimensions, rootlet proportion relative to the spinal cord, gray and white matter dimensions. Additionally, we used Friedman’s test to compare measurements across four anatomical locations (dorsal, ventral, lateral left, and lateral right). When a significant effect was detected, a *post-hoc* pairwise comparison with Bonferroni correction was performed to identify specific differences between anatomical locations. Data are presented as mean ± standard deviation, and a significance threshold of *p* < 0.05 was used for all statistical comparisons. Analyses were conducted using SPSS Statistics 20.0 (IBM Corp., Armonk, NY, United States).

## 3 Results

In the present study, the primary objective was to develop a high-resolution neuroanatomical model of the feline spinal cord. Here, we present data sequentially, layer by layer, from the outermost to the innermost structures of the spinal cord.

### 3.1 Dura mater dimensions and organization

**Figure 2:**
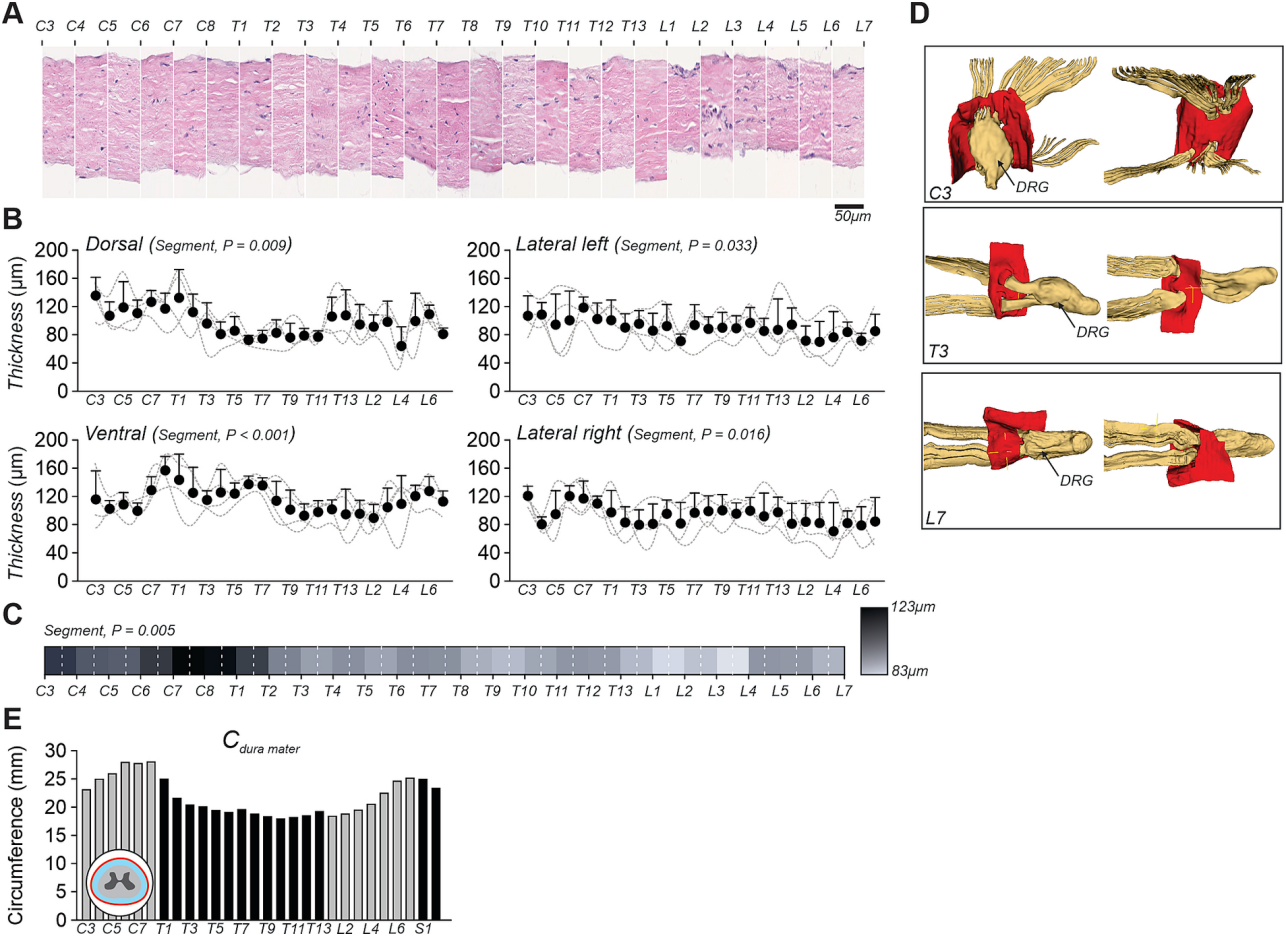
Dura mater analysis along the feline spinal cord. **(A)** Histological sections of the dura mater from spinal segments C3 to L7, stained with hematoxylin and eosin. **(B)** Thickness measurements of the dura mater in four anatomical orientations (dorsal, ventral, lateral left, and lateral right), across spinal segments. Each data point indicates the mean ± SD for the group (*n* = 4 cats). Dashed lines indicate individual data. *p-*values comparing spinal segments are indicated (Friedman’s test). **(C)** Grayscale representation of average dura mater thickness per segment for the group (*n* = 4 cats), where darker shades correspond to greater thickness. **(D)** 3D reconstructions of the dura mater (in red) and dorsal root ganglia (DRG) at three representative spinal levels: C3, T3, and L7. **(E)** Circumference measurements of the dura mater computed from a 3D model reconstructed from MRI and Micro-CT imaging of one cat.

To determine variations in dura mater along the spinal cord, we measured its thickness from C3 to L7 (Figure 2). Histological sections predominantly revealed a unique fibrous layer composed of wavy collagen fibers, arranged in an organized manner with dispersed fibroblasts (Figure 2A). In some cases, a second layer, potentially corresponding to the dural border cell layer, can be distinguished. These observations contrast with the dura mater of the brain, which is known to exhibit three well-defined layers (Kinaci et al., 2020). At this stage, identifying a consistent pattern in the appearance of this second layer across spinal levels remains challenging. Further investigations, including additional histological staining are currently underway to refine these observations.

We next explored dura mater thickness in four anatomical regions (dorsal, ventral, left lateral, and right lateral) from C3 to L7 across cats (Figure 2B). Dura mater thickness ranged from 36.1 to 172.3 μm in the dorsal region, 49.3–178.8 μm in the ventral region, 35.8–151.8 μm in the left lateral region, and 34.0–145.0 μm in the right lateral region. Visually, thickness fluctuated more prominently in the ventral and dorsal regions compared to the lateral sides. In the lateral regions, dura mater thickness appeared to gradually decrease caudally. We found a significant difference between the four anatomical regions (*p* = 0.038). On average, the thickness appeared greater on the ventral side (114.52 μm ± 26.73) compared to the dorsal side (97.46 μm ± 28.41), followed by the right lateral (92.44 μm ± 25.55) and left lateral (90.32 μm ± 25.44) sides. *Post hoc* analysis revealed significant differences between left and ventral sides (*p* = 0.006), and right and ventral sides (*p* = 0.028), while all other pairwise comparisons were non-significant (left and right, *p* = 0.584; left and dorsal, *p* = 0.273; right and dorsal, *p* = 0.584; dorsal and ventral, *p* = 1.00). Dura mater thickness significantly differed across spinal segments. These data indicate that dura mater thickness varies depending on anatomical side and spinal segment.

Figure 2C provides a consolidated view of the total mean dura mater thickness across spinal segments (C3–L7), represented using a grayscale gradient, where darker and lighter shades indicate greater and thinner thickness, respectively. We found a significant difference in thickness across spinal segments. On average, the spinal dura mater thickness ranged between 83 and 123 μm, with the greatest thickness observed at C6–C7 (122.62 μm ± 5.84) and the thinnest at L3–L4 (80 μm ± 20.1). Spinal dura mater thickness is known to be thinner than in the brain, which is approximately 200 μm in cats (Kinaci et al., 2020).

Figure 2D illustrates a segmentation of the dura mater (red) at three spinal levels (C3, T3, and L7), providing a spatial representation of its position relative to the DRG. Our observations indicate that the spinal dura mater terminates just before the DRG across all spinal segments.

To further characterized dura matter characteristics, we also quantified the dural circumference on the 3D model combining MRI and Micro-CT data in one cat (Figure 2E). This measurement revealed maximal values at the cervical enlargement (C6–C8, ~28 mm) and at the lumbosacral junction (L7–S1, ~25 mm), while the thoracolumbar segments (T3–L4) exhibited narrower circumferences, averaging around 20 mm.

### 3.2 DRG dimensions

**Figure 3:**
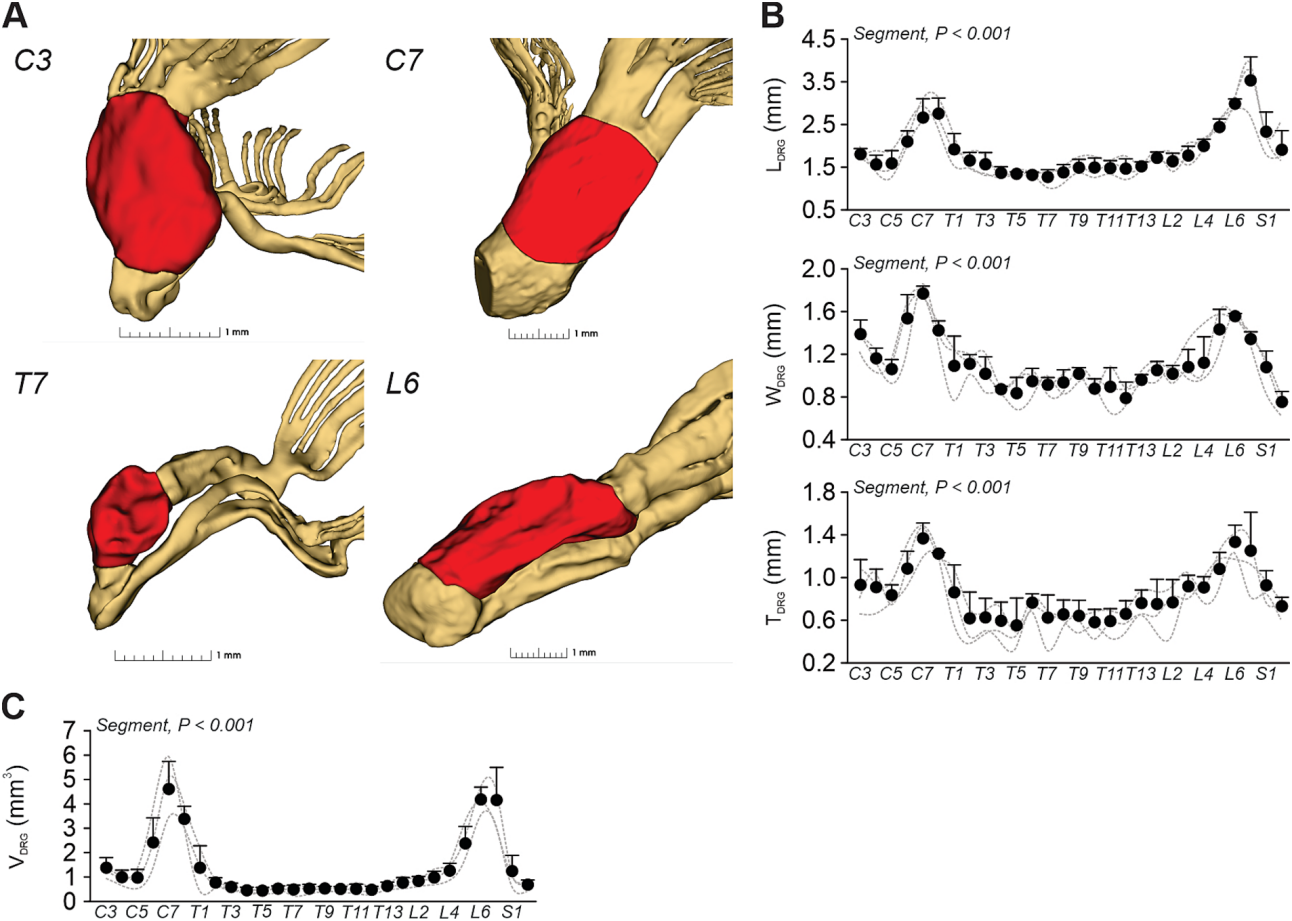
Morphometric analysis of dorsal root ganglia. Morphometric parameters were extracted from high-resolution micro-CT data. **(A)** Representative 3D renderings of segmented DRG (red) at four spinal segments: C3, C7, T7, and L6, illustrating their anatomical position and shape relative to the rootlet. **(B)** Segment-by-segment quantitative measurements of DRG length (L_DRG_), width (W_DRG_), and thickness (T_DRG_) from C3 to S2. **(C)** Computed volume (V_DRG_) of DRG across spinal levels. Each data point indicates the mean ± SD for the group (*n* = 4 cats). Dashed lines indicate individual data. *p*-values comparing spinal segments are indicated (Friedman’s test).

The DRG, located bilaterally along the spinal cord, house the cell bodies of primary afferent neurons responsible for transmitting somatosensory and nociceptive information to the central nervous system. Figure 3A illustrates the segmentation of DRG (red) at four segments (C3, C7, T7, and L6), revealing morphological variations that reflect the pattern observed in humans, where DRG are generally larger in the cervical and lumbar regions (Haberberger et al., 2019). Figure 3B quantifies these variations, illustrating changes in length (L_DRG_), width (W_DRG_), and thickness (T_DRG_) from C3 to S1. For Cat 4, measurements at C8 and T1 could not be obtained due to tissue damage during dissection. Consequently, statistical analysis was performed on three cats from C3 to S2, demonstrating significant differences in L_DRG_, W_DRG_, and T_DRG_ across spinal segments. Notably, we observed two enlargements at C5–C8 and L5–L7. The longest lengths were recorded at C8 (2.76 ± 0.37 mm) and L7 (3.53 ± 0.55 mm) in the cervical and lumbar regions, respectively. The maximum width and thickness were observed at C7 (1.77 ± 0.07 mm; 1.37 ± 0.14 mm, respectively) and at L6 (1.56 ± 0.02 mm; 1.33 ± 0.16 mm, respectively) in the cervical and lumbar regions, respectively. In contrast, DRG in the thoracic region (T2–T13) exhibited consistently smaller dimensions, aligning with their primary role in trunk innervation.

We observed significant DRG volume (V_DRG_) differences across spinal segments, following a distribution consistent with previous measurements, with the highest volumes found at lower cervical (C6–C8, ~2.5–4.8 mm^3^) and lumbar (L5–L7, ~2.5–4.1 mm^3^) segments (Figure 3C).

### 3.3 Rootlets dimension and organization

**Figure 4:**
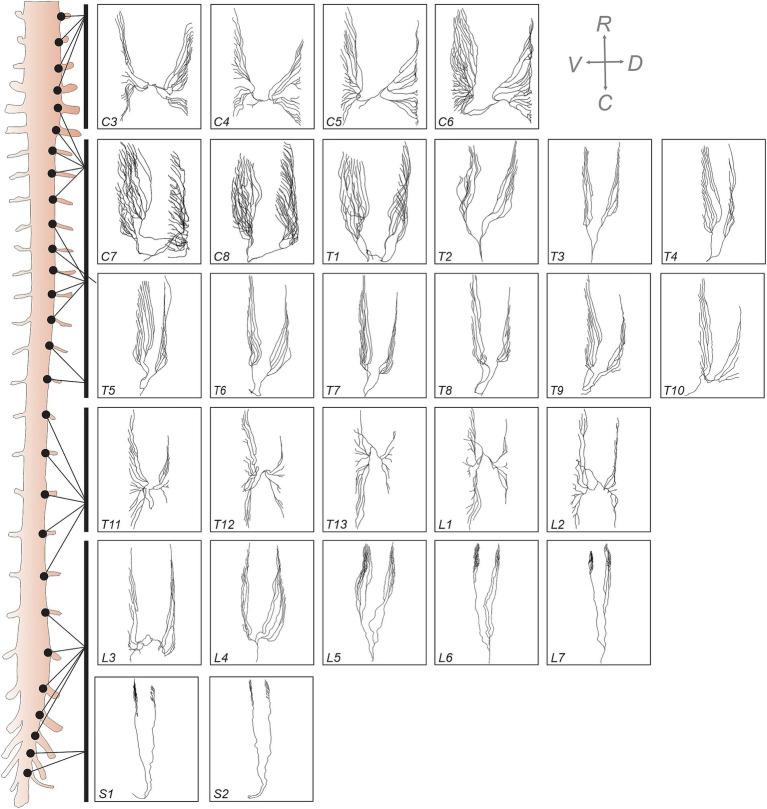
Comprehensive representation of rootlet organization along the feline spinal cord. Left panel: Schematic overview of the feline spinal cord indicating the location of each spinal segment from C3 to S2. Right panel: 3D reconstructions of individual dorsal and ventral rootlets segmented from high-resolution micro-CT imaging at each spinal level. Each panel highlights the characteristic distribution and orientation patterns of the rootlets for that specific segment. Anatomical directions are indicated in the top-right inset (R: rostral, C: caudal, V: ventral, D: dorsal).

By leveraging high-resolution imaging, we investigated morphological characteristics, architectural organization, and proportional distribution of dorsal and ventral rootlets across spinal segments. Figure 4 presents a comprehensive anatomical reconstruction of spinal rootlet arborization along the spinal cord, emphasizing regional variations in rootlet distribution and orientation. The left panel provides a schematic representation of the spinal cord, while the right panels present individual rootlet tracings for each segment from C3 to S2, offering a comparative visualization of rootlet expansion patterns across the spinal cord for one cat. In the upper cervical region (C3–C5), rootlets extend in a rostro-caudal direction, forming a widespread, dispersed architecture, likely to maximize sensory and motor innervation coverage. Moving caudally, the lower cervical segments (C6–C8), where the brachial plexus emerges (Emamhadi et al., 2016), rootlets became progressively denser and more clustered, exhibiting a more condensed and interwoven pattern. From C7 to T10, rootlets exhibited an arciform, rostrally oriented organization, maintaining a linear, streamlined configuration along the spinal cord. From T10 to L2, rootlets began to re-expand laterally, resembling the bilateral arborization pattern observed in upper cervical segments. Notably, the ventral surface area covered by rootlets appeared larger than the dorsal surface. Finally, in the lumbosacral region (L3–S2), rootlets again adopt an arciform organization.

Table 1 documents the number of rootlets observed in both the dorsal and ventral regions across spinal segments in one cat, as the high-resolution images from the other three cats did not allow for sufficiently precise quantification. The data revealed variations in the number of rootlets across segments, with higher numbers at cervical and lumbar enlargements. Interestingly, we also observed a difference in the number of rootlets between dorsal and ventral regions. Specifically, at C3, the number of dorsal rootlets was higher compared to the ventral side. At C4, C5, T3, T8, T9, T13, L3, L4, L7, and S2, the dorsal and ventral rootlet counts were similar. However, for C6 to T2, T4 to T7, T10, T11, L1, L2, L5, L6, and S1, ventral rootlet counts were visibly higher, consistent with greater numbers of motor/efferent axons.

**Figure 5:**
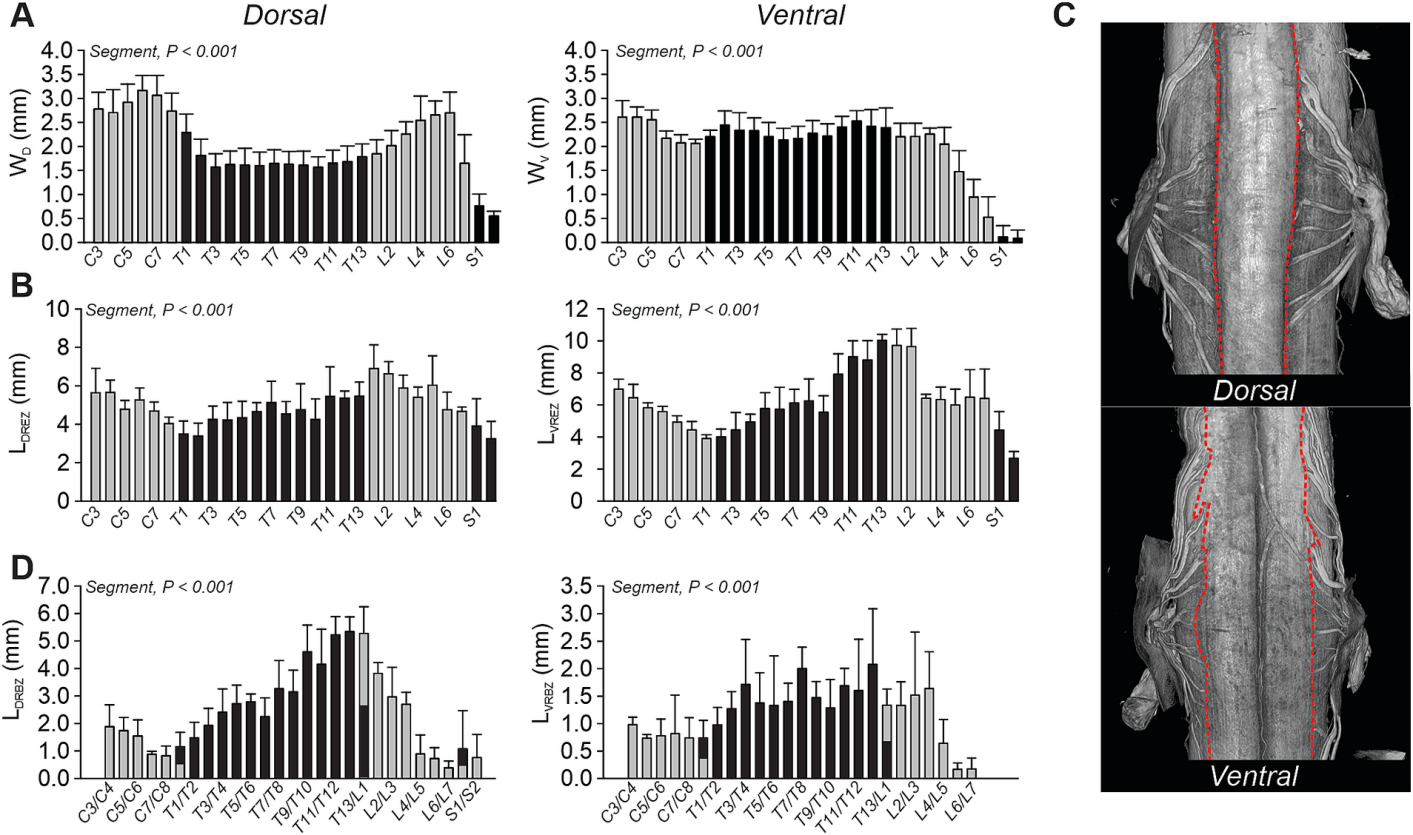
Quantitative analysis of dorsal and ventral rootlet organization along the feline spinal cord. **(A)** Bar plots showing the width of the dorsal (W_D_, left panel) and ventral (W_V_, right panel) columns across spinal segments. **(B)** Bar plots showing the rostrocaudal length of the dorsal (L_DREZ_, left panel) and ventral (L_VREZ_, right panel) rootlet entry zones across segments. **(C)** Representative dorsal and ventral views of the spinal cord highlighting the rootlet entry zones (red dashed lines). **(D)** Bar plots showing the interzone distance between dorsal (L_DRBZ_, left panel) and ventral (L_VRBZ_, right panel) rootlet clusters across segments. Each measurement was obtained from MicroCTex reconstructions. Data are presented as mean ± SD for the group (*n* = 4 cats). *p*-values comparing spinal segments are indicated (Friedman’s test).

To investigate morphological variations of the rootlets, we measured the width of the dorsal (W_D_) and ventral (W_V_) columns, the lengths of the dorsal (L_DREZ_) and ventral (L_VREZ_) rootlet entry zones, and the distances between these zones on both the dorsal (L_DRBZ_) and ventral (L_VRBZ_) aspects across different spinal segments (Figure 5). Both column widths exhibited significant variations across spinal segments (Figure 5A). In the cervical region, W_D_ increased progressively, peaking at C6–C7 (~3.0 mm). Caudal to C8, W_D_ decreased, reaching a plateau between T3 and T12 (~1.5 mm). We then observed a gradual increase from T13 to L6, with a second peak at L6 (~2.5 mm), followed by a decline to ~0.5 mm at S2. Interestingly, W_V_ exhibited minimal variations between C3 and L4 (~2.5 mm) before decreasing considerably from L5 and more caudally, being nearly absent at S1–S2 (~0.1 mm).

Regarding the lengths that characterize the rootlet span as they enter the spinal cord, we observed significant segmental variations for both dorsal and ventral entry zones (Figure 5B). L_DREZ_ and L_VREZ_ decreased progressively from C3 to T2, then increased from T2 to L1/L2, followed by another decline more caudally. Across spinal segments, the ventral rootlets covered a significantly greater rostrocaudal distance than the dorsal rootlets (L_DREZ_, 4.89 ± 0.57 mm; L_VREZ_, 6.26 ± 0.45 mm; *p* = 0.041). These findings suggest that motor rootlets cover a larger portion of the spinal cord. We also observed that dorsal rootlets entered the dorsal columns in an organized linear pattern, while ventral rootlets exited the ventral columns less clearly and without a distinct demarcation (Figure 5C).

To quantify the portion of the spinal cord not covered by rootlets, we measured the inter-rootlet distance between adjacent rootlets in the dorsal (L_DRBZ_) and ventral (L_VRBZ_) regions (Figure 5D). These both significantly varied across spinal segments. L_DRBZ_ progressively decreased from C3 to C8 before increasing from T1 to T13 followed by a decline more caudally. These results indicate that, at cervical (C6–C8) and lumbar (L4–L7) enlargements, the dorsal and ventral spinal cord is almost entirely covered by rootlets, whereas at thoracic levels, there are significantly larger gaps between two adjacent segments. L_VRBZ_ followed a similar trend as L_DRBZ_, but ventral inter-rootlet distances were significantly shorter than dorsal distances (L_DRBZ_, 2.57 ± 0.43 mm; L_VRBZ_, 1.19 ± 0.16 mm; *p* = 0.046). This reflects a denser coverage of the ventral cord by rootlets. At L7–S2, ventral rootlets exhibited significant overlap, making precise measurements difficult based solely on raw Micro-CT images.

**Figure 6:**
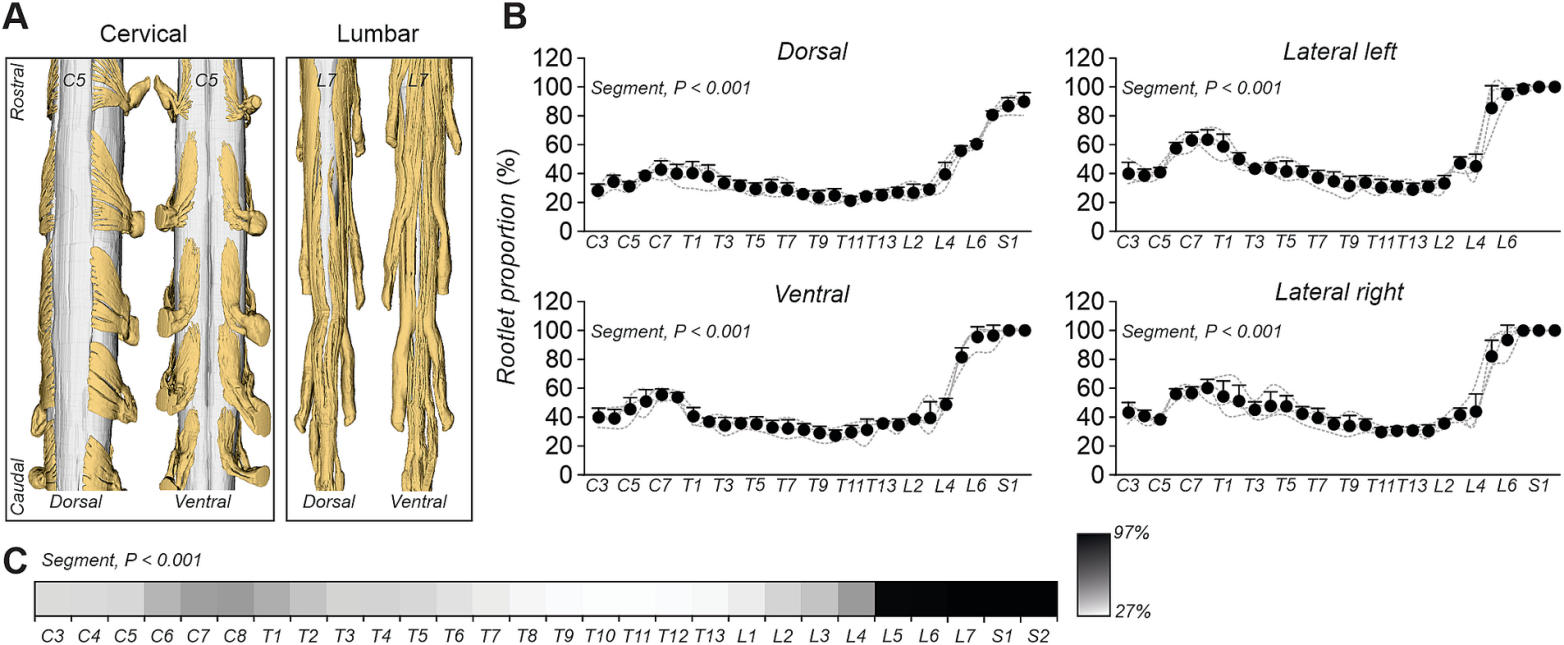
Quantification of rootlet coverage relative to the spinal cord surface. **(A)** 3D reconstructions of dorsal and ventral rootlets in C5–T1 and L7–S2 segments, showing distinct spatial and surface coverage patterns. **(B)** Segmental quantification of rootlet surface proportion in four anatomical views (dorsal, ventral, left lateral, and right lateral) expressed as a percentage of total visible spinal cord surface. Each data point indicates the mean ± SD for the group (*n* = 4 cats). **(C)** Summary of rootlet proportion by spinal segment, displayed using a grayscale heat map, where darker shades indicate higher rootlet coverage. In B and C, *p-*values comparing spinal segments are indicated (Friedman’s test).

At the caudal segments of the cervical and lumbar cord, dorsal and ventral rootlets covered a greater portion of the spinal cord compared to white matter (Figure 6A). Rootlet proportion varied significantly with spinal segments in the four anatomical regions studied (Figure 6B). The data revealed a first peak at lower cervical segments (50–60%), a smaller mainly constant proportion (25–34%) from T6–L2 before increasing sharply from L5 to S2, where rootlets covered most of the cord in all four regions. Across the four regions, rootlet proportion varied significantly from C3 to S2, ranging from 27 to 97% (Figure 6C). Collectively, these findings underscore region-specific variations in rootlet distribution, with increased coverage at spinal enlargements.

### 3.4 Gray and white matter and cross-sectional dimensions

**Figure 7:**
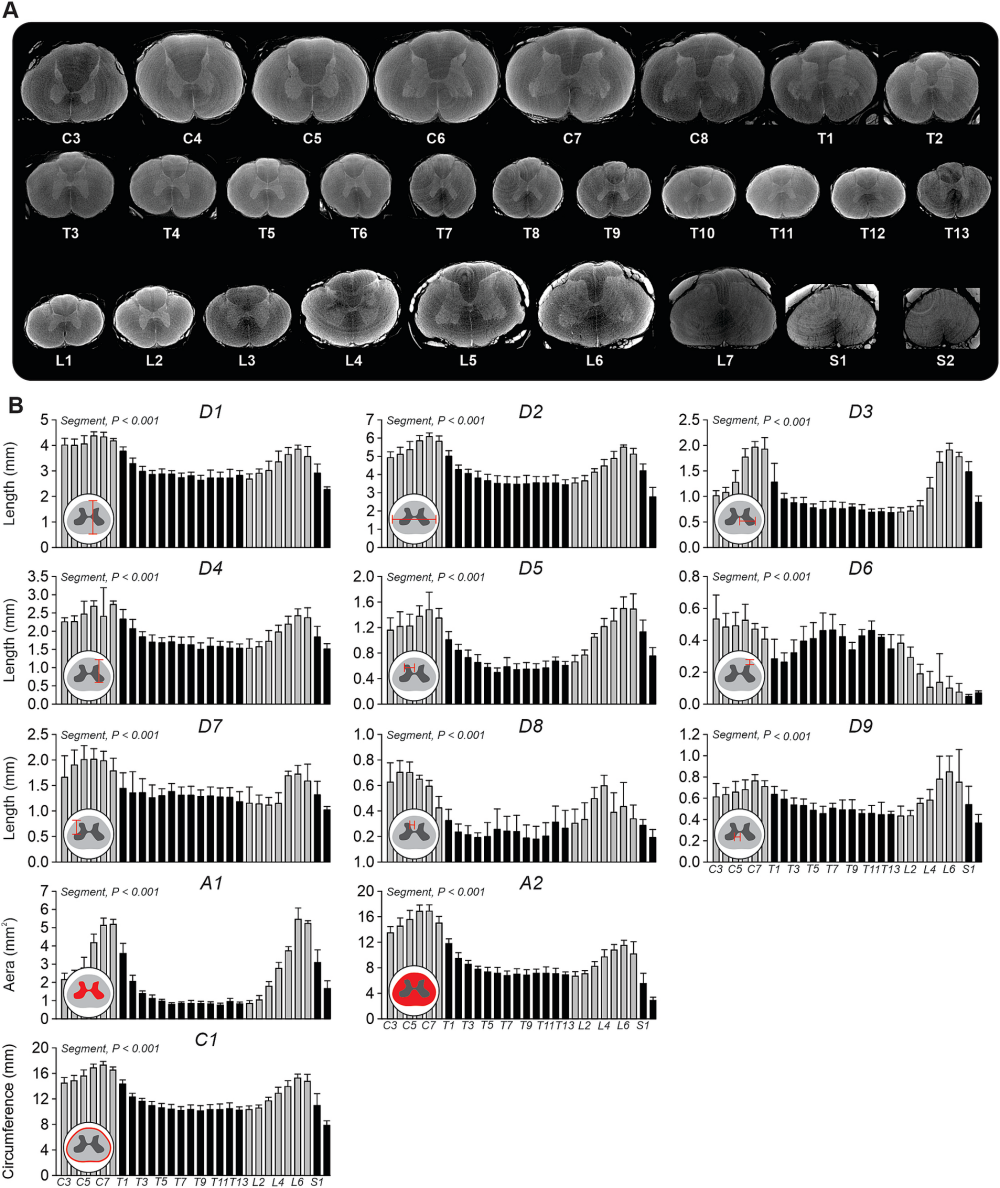
Cross-sectional analysis of white and gray matter along the feline spinal cord. **(A)** Representative axial views of high-resolution micro-CT images showing spinal cord morphology from segment C3 to S2 in one cat. **(B)** Quantitative measurements of morphometric parameters across spinal segments, including spinal cord height (D1), width (D2), mediolateral (D3, D5, D8, D9) and depth (D4, D6, D7) distances. Gray matter area (A1), white matter area (A2), and white matter circumference (C1) were also computed to capture cross-sectional structural variations. Insets illustrate measurement definitions and anatomical landmarks used for extraction. Data are presented as mean ± SD for the group (*n* = 4 cats). *p*-values comparing spinal segments are indicated (Friedman’s test).

We measured the morphological characteristics of the gray and white matter in the spinal cord to illustrate how these structures vary along its length (Figure 7). In the cervical region, we observed the largest cross-sectional areas in the cervical enlargement (C5–C8), as expected (Figure 7A). The size of the gray matter (i.e., the H-shape portion) was visibly larger at more rostral cervical segments. At thoracic segments, the cord was smaller with a small gray matter, particularly in the ventral horn. Then, we observed a gradual increase in spinal cord size from L1 to L7, marking the lumbar enlargement responsible for innervating hindlimb muscles. The gray matter expanded significantly, with large ventral horns. In the sacral region, spinal cord size diminished, and the contrast between gray and white matter became less distinct.

To quantify rostro-caudal variations, we measured several parameters, including white matter height (D1) and width (D2), ventral horn lateral (D3) and medial (D9) distances from the midline, ventral (D4) and dorsal (D7) depths from the dorsal surface, and dorsal horn lateral (D5) and medial (D8) distances from the midline, as well as dorsal depth (D6) (Figure 7B). All measured parameters significantly varied across spinal segments. D1 reached its highest values in the cervical enlargement (C5–C8, ~4.2–4.6 mm) and at L6 (~3.6–4.1 mm), with a reduction in thoracic and upper lumbar segments (~2.8 mm at T3–L2). Similarly, D2, peaked at C5-C8 (~5.9–6.4 mm) and L6 (~5.5 mm), while thoracolumbar levels (T4–L2) exhibited lower values (~3.6 mm). D3 through D9 exhibited two peaks at cervical and lumbar enlargements, except for D6, which showed a decrease in these regions. To complement these linear measurements with cross-sectional data, we also evaluated the areas of gray matter (A1) and white matter (A2). A1 had peak values at C7–C8 (~4.9–5.9 mm^2^) and L6–L7 (~5–6.1 mm^2^) with marked reductions at thoracic and upper lumbar segments (~1.0 mm^2^ at T3–L2). A2 followed the same distribution, with the largest areas observed at lower cervical (C6–C7, ~15.5–18.4 mm^2^) and lumbar (L6–L7, ~10–12.7 mm^2^) segments.

Measurement C1, representing white matter circumference at each spinal segment, peaked at C6–C8 (~16.5–17.3 mm) and L6–L7 (~14 mm), while significantly lower values were observed across thoracic and upper lumbar segments (~10.5 mm at T4–L2).

### 3.5 Cerebrospinal fluid volume and dispersion

**Figure 8:**
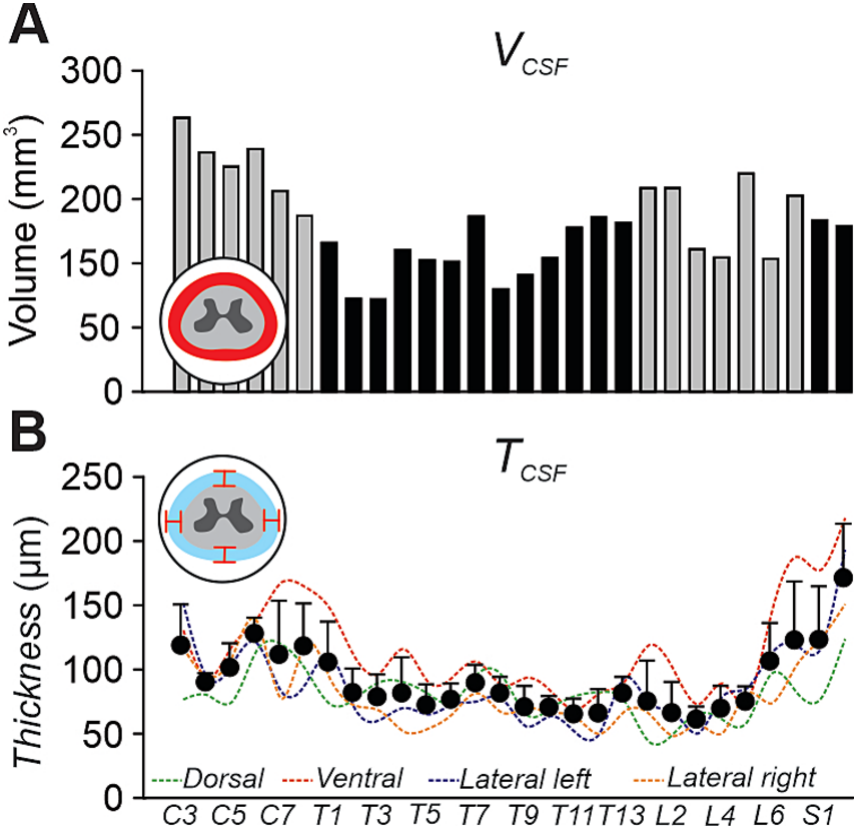
Quantification of cerebrospinal fluid (CSF) morphological features along the spinal cord. **(A)** Bar graph showing segmental variations in CSF volume from C3 to S1, extracted from a co-registered MRI and MicroCTex dataset in one cat. **(B)** Graph showing CSF thickness in one cat. Colored dashed lines represent individual anatomical positions (dorsal, ventral, lateral left, and lateral right), while black dots indicate the average thickness per segment across all positions as mean ± SD. Insets illustrate the location and orientation of each measurement within the spinal canal.

We characterized morphological properties of the (CSF) by measuring both its volume (Figure 8A) and thickness (Figure 8B) on the 3D model by combining MRI and MicroCTex data. The CSF volume varied along the spinal axis, ranging from 100 to 260 mm^3^ depending on the segment, with a total spinal CSF volume of approximately 4.40 mL (4401.89 mm^3^) between C3 and S2. CSF thickness, measured at four anatomical positions (dorsal, ventral, left and right lateral), fluctuated with spinal level and ranged between 60 and 160 μm.

### 3.6 Three-dimensional model and its compatibility with computational software

To provide the scientific community with a comprehensive resource for advanced morphological analysis and computational modeling, we developed a high-resolution 3D model of the spinal cord and its surrounding structures, integrating vertebrae, dura mater, CSF, rootlets, dorsal root ganglia, white matter, and gray matter from multimodal imaging datasets, including MRI, high-resolution Micro-CT (MicroCTpm and MicroCTex), from C3 to S3 in one cat. This female cat was 466 days old, weighed 3.35 kg, and measured 378.13 mm from the base of the skull to the base of the tail.

**Figure 9:**
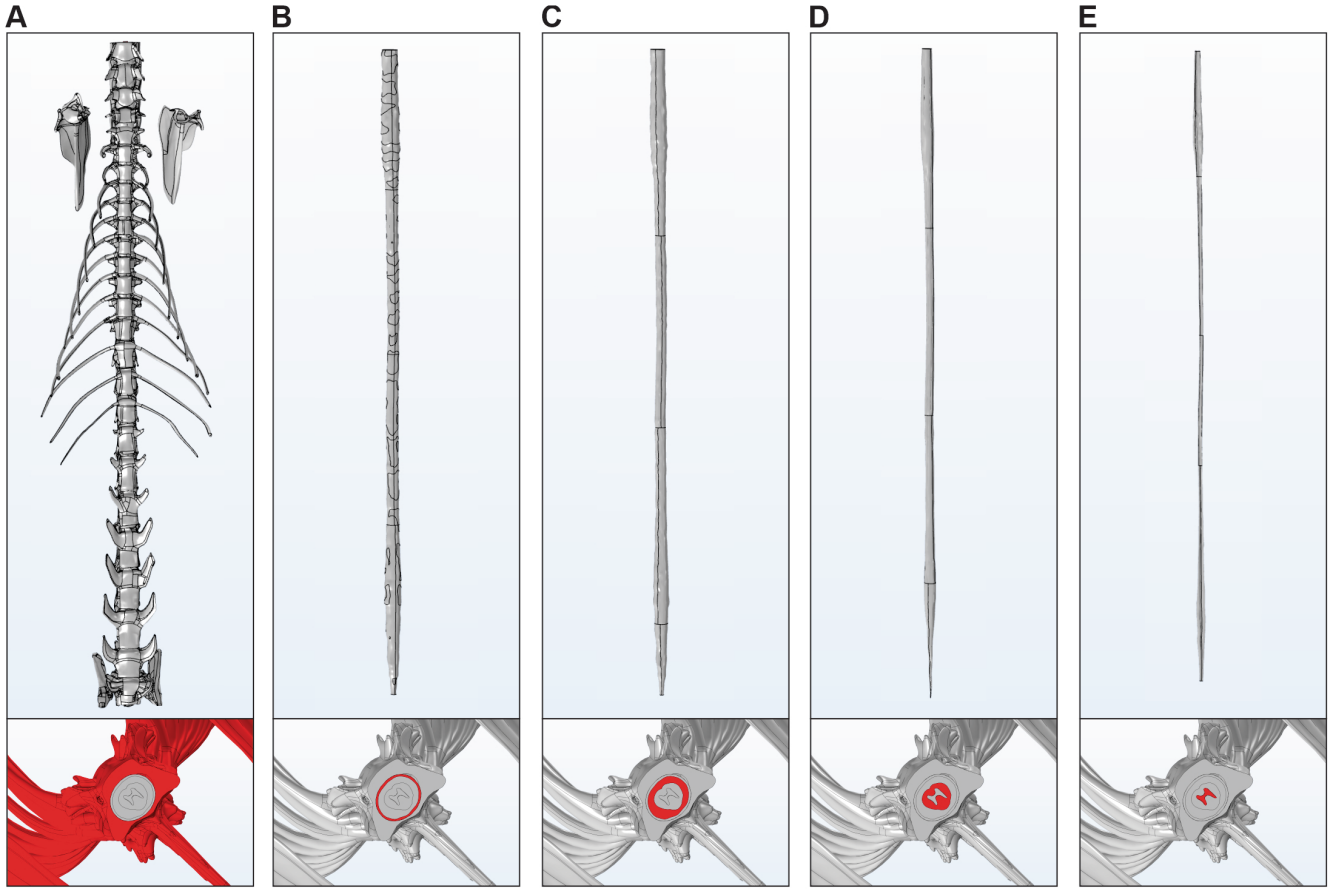
3D model of the feline spinal cord for COMSOL simulation. The 3D model includes the vertebrae **(A)**, dura mater **(B)**, cerebrospinal fluid **(C)**, white matter **(D)**, and gray matter **(E)**. Insets at the bottom of panels B–F display transverse cross-sections with the segmented structure highlighted in red, illustrating its anatomical location and contour.

**Figure 10:**
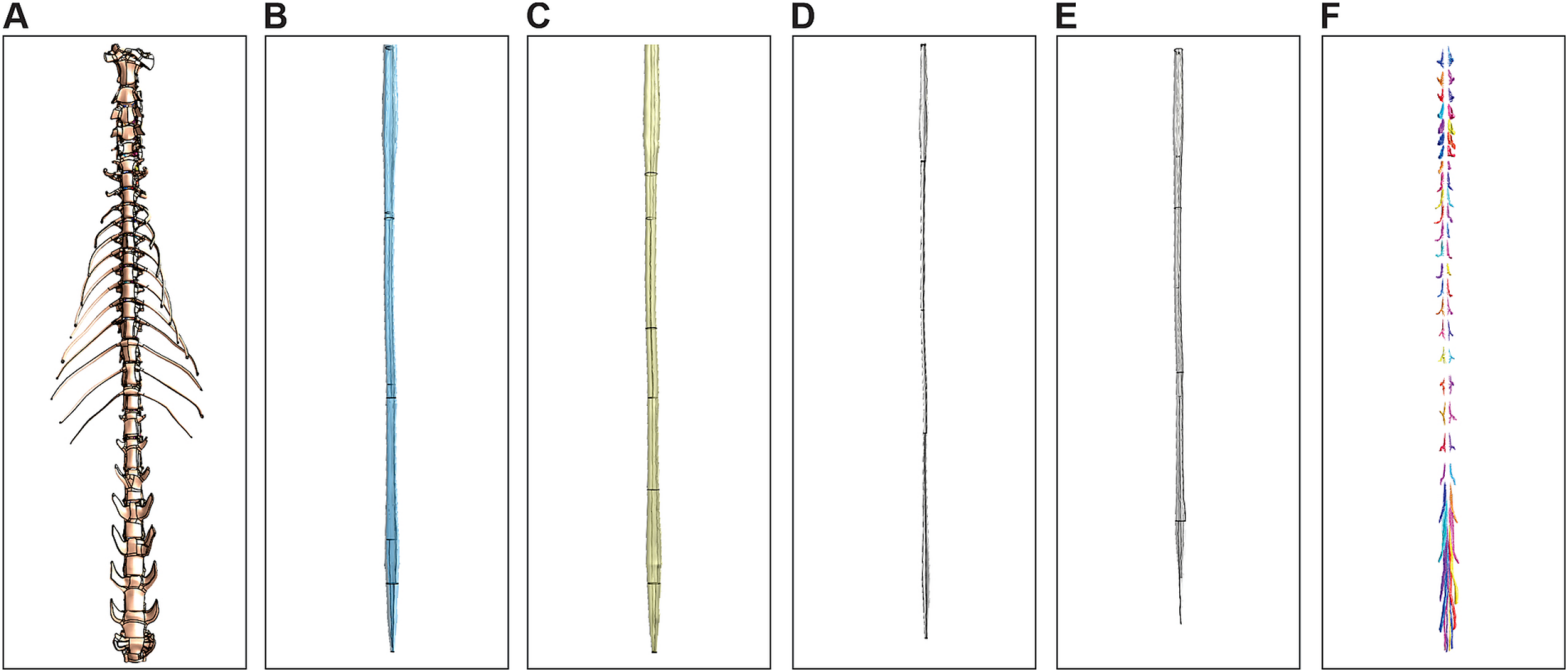
Multi-tissue 3D model of the feline spinal cord imported into Sim4Life. Segmented model components displayed individually: vertebrae **(A)**, dura mater **(B)**, cerebrospinal fluid **(C)**, white matter **(D)**, gray matter **(E)**, and **(F)** rootlets and DRG.

To evaluate the operational use of our model in computational frameworks, we tested its integration with two commonly used simulation platforms: COMSOL (v5.5, COMSOL AB, Stockholm, Sweden) and Sim4Life (v7.2.4, ZMT Zurich MedTech AG, Zurich, Switzerland). Figures 9, 10 presents the different layers of the model within COMSOL and Sim4Life, respectively. The 3D model includes the vertebrae (A), dura mater (B), CSF (C), white matter (D), and gray matter (E). Rootlets were excluded from the model due to COMSOL’s meshing limitations. Specifically, the automatic geometry unification process within the software led to major self-intersecting edges and faces, rendering the meshing of rootlets unfeasible. In contrast, the structured meshing approach used by Sim4Life successfully processed the entire model, including the rootlets (Figure 10F).

Table 2 summarizes the computational demands for each software, highlighting the difference in the number of elements, physical memory usage, and time to open. While COMSOL required over 3.5 GB of memory and nearly 15 min of processing to mesh the model without rootlets, Sim4Life processed the full model in under a minute using less than 1 GB of memory. We initially attempted meshing in Sim4Life using its unstructured mesher (comparable to COMSOL’s meshing approach), but this method proved computationally prohibitive, requiring excessive memory and runtime. Consequently, we adopted Sim4Life’s structured meshing, which produced a high-quality mesh efficiently. These findings demonstrate that our 3D spinal cord model is computationally viable, particularly in Sim4Life, and can serve as a foundation for advanced simulations.

**Table 2:** Compatibility of our model.

Model	Software	Meshing type	No elements	Physical memory (GB)	Time (s)
Gray and white matter, CSF, Dura, Vertebrae	COMSOL	Unstructured	3.25^07^	3.57	873.92
Full model Full model	Sim4Life Sim4Life	Structured unstructured	1.29^09^ 1.29^09^	0.95 > 64	54.86 >259200*

*Meshing did not complete due to memory limitations. The table shows the number of mesh elements, physical memory usage (in GB), and simulation time (in seconds) for two configurations: (1) a segmented model including gray matter, white matter, cerebrospinal fluid (CSF), dura mater, and vertebrae in COMSOL, and (2) the full anatomical model in Sim4Life.

## 4 Discussion

By combining high-resolution Micro-CT with Lugol’s Iodine staining, we visualized fine anatomical structures that are typically too small to be seen with conventional imaging techniques. We then segmented these structures and integrated them with MRI and standard CT datasets to generate a comprehensive and anatomically detailed 3D model of the feline spinal cord.

### 4.1 Morphological parameters

In the present study, we found that the thickness of the spinal dura mater varied more prominently in the dorsal and ventral regions compared to the lateral sides, ranging from 83 to 123 μm across spinal segments (Figure 2C). The dura mater is composed of a dense connective tissue layer primarily composed of collagen fibers and fibroblasts, which confer its strength and mechanical resilience (Kinaci et al., 2020; Yu et al., 2013). Thickness varied more prominently in the dorsal and ventral regions compared to the lateral sides, though no significant differences were found between dorsal and ventral measurements. In humans, the thickness of the spinal dura mater also varies across spinal segments (Cavelier et al., 2022a; Hong et al., 2011; Kwon et al., 2018). For instance, it measures approximately 260 μm in the cervical region, 224 μm in the thoracic region, and 159 μm in the lumbar region (Cavelier et al., 2022a). However, other studies have reported slightly higher values at cervical and thoracic levels (Hong et al., 2011; Kwon et al., 2018). Discrepancies between studies likely arise from different methodological and analytical techniques. In landrace cross pigs, the dura mater also varies depending on the spinal segments (Cavelier et al., 2022b), as well as between the dorsal and ventral sides, although conclusions differ among studies (Cavelier et al., 2022b; Chin et al., 2024). At the thoracic level in pigs, thickness ranges from 90 μm to 134 μm, depending on the study. Functionally, greater thickness may indicate increased mechanical protection against trauma, particularly at cervical enlargements (Nagel et al., 2018). Furthermore, from a biophysical standpoint, a thicker dura also contributes to greater electrical insulation (Sin and Coburn, 1983). As such, this parameter is particularly important to consider when conducting computational simulations or designing neuromodulation protocols targeting the spinal cord (Holsheimer, 1998).

Between the dura mater and the spinal cord flows the CSF, which serves essential physiological roles by cushioning the spinal cord, delivering nutrients, and clearing metabolic waste (Czarniak et al., 2023). Using our 3D model reconstructed from MRI and MicroCTex datasets, we were able to estimate the total spinal CSF volume and its morphological characteristics at each spinal segment in one animal. The thickness of the CSF layer varied across spinal segments, ranging on average from 61 to 171 μm. We found a total spinal CSF volume of approximately 4 mL in the cat. In humans, the spinal CSF volume ranges between 81 and 110 mL, depending on the methodology and population studied (Edsbagge et al., 2011; Strbačko et al., 2024). Regional variations have also been described, with the cervical region containing ~19 mL of CSF, the thoracic region ~38 mL, and the lumbosacral region ~25 mL (Edsbagge et al., 2011). The CSF can play an important role in the effectiveness of spinal stimulation protocols. A thicker CSF layer increases the distance between the electrode and the spinal cord, thereby reducing stimulation efficiency, raising activation thresholds, and increasing current spread (Holsheimer et al., 1995; Huang et al., 2014). Moreover, it is well established that spinal CSF volume and geometry vary with body position (e.g., supine, prone, lateral). Studies have shown that spinal cord and CSF dynamics are posture-dependent, with ventral or dorsal shifts of the cord occurring based on gravity and spinal curvature (Magnaes, 1989; Strbačko et al., 2024). It is also important to note that during epidural electrode implantation, the device inevitably compresses the dura mater toward the spinal cord, thereby reducing CSF thickness underneath the electrode. These shifts alter the electrode-to-cord distance, thus modifying activation thresholds and stimulation selectivity (Holsheimer, 1998; Holsheimer and Wesselink, 1997).

On either side of the spinal cord, the DRG house cell bodies of primary afferent neurons responsible for transmitting peripheral sensory inputs to the central nervous system. In cats, there are 36 pairs compared to 31 in humans (Haberberger et al., 2019). In this study, we found significant variations in DRG size along the spinal cord in cats, with prominent enlargements at cervical (C5–C8) and lumbar (L5–L7) levels (Figure 3B). The longest DRG were measured at C8 (2.76 ± 0.37 mm) and L7 (3.53 ± 0.55 mm), while maximum width and thickness were found at C7 (1.77 ± 0.07 mm; 1.37 ± 0.14 mm) and L6 (1.56 ± 0.02 mm; 1.33 ± 0.16 mm), respectively. Conversely, DRG in the thoracic segments were consistently smaller, reflecting their primary role in innervating the trunk rather than the limbs. In humans, DRG morphology also varies across spinal segment (Haberberger et al., 2019), but much of the existing research has focused on the lumbar region. For example, in humans, both the width and length of DRG gradually increase from rostral to caudal lumbar segments, ranging from 4.36 mm (length) and 5.39 mm (width) at L1 to 5.82 mm and 10.83 mm at L5, respectively. DRG have emerged as promising targets for neuromodulation to evoke motor responses after spinal cord injury in cats (Urbin et al., 2019) and humans (Soloukey et al., 2020, 2021). In humans, stimulation of the L4 DRG induces robust knee extension, enabling assisted weight-bearing and demonstrating selective activation of quadriceps muscles (Soloukey et al., 2021). This level of specificity highlights the potential utility of targeting DRG in sensorimotor recovery strategies.

From the DRG, somatosensory signals are relayed into the spinal cord via dorsal rootlets, while ventral rootlets emerge directly from the spinal cord to carry motor outputs. In the present study, we observed that in upper cervical (C3–C4) and thoracolumbar (T10–L2) regions, rootlets were bilaterally distributed and displayed a widespread pattern (Figure 4), likely to enhance sensory and motor innervations. In contrast, lower cervical (C5–C8) and lumbosacral (L3–S2) segments showed dense, clustered, arciform rootlet arrangements, suggesting specialized connectivity for limb control. We also found that rootlet number varied across spinal segments, peaking at cervical and lumbar enlargements. Interestingly, cats had more ventral than dorsal rootlets, particularly in these enlargements, suggesting a potentially higher motor fiber density. Moreover, our measurements of dorsal and ventral column widths across segments showed patterns closely matching those in humans, with peaks in cervical and lumbar enlargements. Similarly, the dorsal and ventral rootlet entry zones showed a progressive decrease from C3 to T2, followed by an increase to L1–L2 and a decline in more caudal segments, again mirroring human findings. As in humans (Mendez et al., 2021), we observed that dorsal rootlets entered the dorsal columns at a very defined line, while ventral rootlets exited the ventral columns without a distinct demarcation.

Lastly, we quantified inter-rootlet distances (L_DRBZ_ and L_VRBZ_), finding dense coverage and minimal spacing at cervical and lumbar enlargements, especially ventrally, where inter-rootlet spacing was significantly shorter than dorsally. In humans, rootlets are difficult to distinguish using MRI, and most anatomical data come from cadaveric dissections, often with variability (Mendez et al., 2021; Zhou et al., 2010). A recent study by Mendez et al. (2021) provided a detailed description of dorsal and ventral roots from C3 to L5 using surgical microscopy in human. They showed that the number of rootlets varied across spinal segments and between dorsal and ventral roots. The greatest number of rootlets was found at the cervical and lumbar enlargements (~8.4 and ~7.9 for dorsal; ~7.2 and ~5.6 for ventral), while thoracic levels had fewer. They measured the distance between the left and right rootlets and found that the width of the dorsal columns (W_D_) was greatest at cervical segments (6.84 mm) and smallest at thoracic ones (5.05 mm), showing a secondary increase in the lumbar region. Conversely, the ventral column width (W_V_) was largest in the cervical region and decreased caudally, reaching its minimum in the sacral segments. The lengths of the dorsal and ventral rootlet entry zones (L_DREZ_ and L_VREZ_), measured as the distance between the most rostral and caudal rootlets at their entry point into the spinal cord, remained relatively stable from C3 to C7, then slightly decreased before increasing again around T4, followed by a progressive decline at L3 and L5.

From a functional perspective, the marked increase in rootlet proportion at the cervical and lumbar levels is particularly significant, as these spinal enlargements are responsible for the complex innervation of the limbs. The higher density of rootlets in these regions likely represents a key adaptation to enhance the efficiency of sensory and motor signal transmission. While epidural stimulation is often described as targeting the spinal cord, our findings suggest that, especially at cervical and lumbar levels, the extensive rootlet coverage over the white matter makes direct activation of the spinal cord unlikely. To illustrate this, we quantified the proportion of rootlets relative to the white matter across spinal segments and observed substantial regional differences, with 50–60% in lower cervical segments and reaching up to 97% from L5 to S2. These anatomical realities must be considered when designing electrical spinal cord stimulation protocols, particularly when aiming for specific outcomes, such as limb activation, bladder function, or postural control. For example, while certain electrode configurations may be effective in mid-thoracic regions, they may require greater spatial precision in the sacral region due to the denser rootlet architecture and compressed geometry of the ventral columns. Furthermore, rootlet orientation should also be considered, as it may critically influence stimulation efficacy.

In the present study, we found gray matter area in the transverse plane peaking at ~5.5 mm^2^ in the cervical (C6–C8) and ~6 mm^2^ in the lumbar (L6–L7) regions, and white matter peaking at ~17 mm^2^ and ~12 mm^2^, respectively. This agrees with histological data from Veshchitskii et al. (2022) in cats. We measured the position of the gray matter relative to the white matter (e.g., distances D3–D9), as in Toossi et al. (2021), but extended this analysis from C3 to S2 (Figure 7B). In humans, the white matter reaches its maximum area (~55 mm^2^) at the cervical enlargement (C4–C5), decreases through the thoracic region (~25–30 mm^2^), and increases again in the lumbar segments (~35–40 mm^2^ at L3–L4), while the gray matter follows a similar but smaller profile (~15–20 mm^2^ at enlargements, <10 mm^2^ in thoracic segments; Henmar et al., 2020).

A new class of electrodes known as circumferential spinal electrodes is emerging as a promising approach to facilitate locomotor recovery through spinal cord stimulation. These devices can be implanted either directly around the spinal cord, as in subdural applications (Woodington et al., 2024), or within the epidural space surrounding the dura mater. In this context, our anatomical data provide valuable guidance for electrode design. Using high-resolution MicroCTex imaging in four cats, we found that spinal cord circumference is greatest at cervical and lumbar levels, which correspond to the spinal enlargements, while it is notably reduced in the thoracic region. These measurements should be interpreted cautiously due to potential sample shrinkage. For electrodes placed circumferentially around the dura mater, we calculated the dural circumference using the 3D model, meaning that shrinkage is not applicable, although the model is not yet fully anatomically accurate (see Limitations section). We observed the same rostrocaudal pattern, with larger circumferences at cervical and lumbar levels and narrower dimensions in the thoracic segments. Dural circumference values ranged from 17.89 to 28 mm across segments (Figure 2E).

### 4.2 Open access 3D model

In this study, we provided a high-resolution, multimodal 3D reconstruction of the feline spinal cord, encompassing key structures such as the vertebrae, dura mater, cerebrospinal fluid, rootlets, dorsal root ganglia, and both white and gray matter. This model integrates data from MRI, post-mortem Micro-CT, and *ex-vivo* high-resolution Micro-CT, co-registered in a common reference space to ensure anatomical consistency. Such detailed anatomical fidelity not only allows for a more comprehensive understanding of spinal architecture but also bridges the gap between experimental and computational research.

Importantly, the compatibility of our model with finite element software such as Sim4Life and COMSOL demonstrates its direct applicability for testing neuromodulation strategies. While COMSOL had limitations in handling the full model with rootlets due to meshing constraints, Sim4Life successfully imported and voxelized the entire model, underscoring its suitability for studies involving electrical field distribution, thermal effects, or bioelectronic interface development.

Moreover, this open-access 3D model can serve as a foundational tool for optimizing electrode placement, designing spinal implants, or developing computational models for injury, recovery, or neuromodulation. By offering detailed morphometric data in a manipulable digital format, it paves the way for more standardized and reproducible research across laboratories working on spinal cord physiology and repair.

### 4.3 Limitations

This study presents limitations that must be acknowledged. First, excision of the spinal cord induces a major release of its natural rostro-caudal tension, likely due to the loss of mechanical continuity with the brainstem and anchoring of the caudal nerve roots. As shown in Table 3, realignment of the *ex-vivo* spinal cord with the intact MRI geometry required a 35–50% linear stretch along the z-axis, indicating substantial shortening after removal from the vertebral canal. In contrast, consistent 10% corrections along the X- and Y-axes across samples most likely reflect isotropic tissue shrinkage introduced during the staining and clearing steps, particularly those involving ethanol and xylene.

**Table 3:** Percentage of linear scaling applied along each spatial axis to match paraffin block dimensions with MRI measurements in one cat.

Spinal level	*X*	*Z*	*Z*
C3-T1	10%	10%	45%
T2-T7	10%	10%	45%
T8-T12	10%	10%	35%
L1-L3	10%	10%	40%
L4-S2	10%	10%	50%

The table shows the percentage of linear transformations applied along the X, Y, and Z axes to align the paraffin-embedded spinal cord volume with the corresponding MRI volume in one cat.

The spinal cord naturally exhibits anatomical curvatures, including cervical and lumbar lordosis. However, to facilitate consistent morphometric analysis, all measurements from high-resolution images were performed on spinal cord volumes that had undergone a straightening transformation. The final 3D model integrating data from MRI, MicroCTpm and MicroCTex was likewise constructed using these geometrically corrected, straightened volumes.

The animal’s positioning during image acquisition is another factor that may have influenced the morphological measurements. In this study, MRI scans were obtained with the cat in a supine position, while MicroCTpm imaging was performed with the animal in a prone position. These two orientations can significantly affect anatomical relationships, particularly the distribution and thickness of the CSF, due to gravitational shifts of the spinal cord within the dural sac.

Although the imaging data used in this study were of high resolution, the process of anatomical segmentation could be subjective, particularly in regions with lower contrast or complex morphology. To minimize inter-operator variability and ensure consistency, the segmentation of all structures including vertebrae, dorsal root ganglia, rootlets, white matter, and gray matter was performed by the same experimenter (Harnie). This approach enhances coherence across datasets but does not fully eliminate the inherent subjectivity associated with manual segmentation.

Anatomical variability of the spinal cord related to age and sex is also expected in cats, as reported in other mammalian species, and may influence overall spinal cord dimensions as well as segmental morphology. However, the limited sample size and the relatively homogeneous characteristics of the animals included in this study did not allow for a systematic assessment of age- or sex-related effects. Future studies including larger and more diverse cohorts will be required to specifically investigate these sources of variability.

The resulting 3D spinal cord model was derived from these anatomical segmentations rather than directly from the documented morphometric measurements. While this may lead to minor differences between measured values and the reconstructed geometry, all geometric components were derived entirely from physiologically grounded imaging data of the same specimen. The model was therefore designed to provide a stable and anatomically coherent framework for spatial reference rather than to replicate exact local dimensions.

## 5 Conclusion

This study presented a comprehensive, high-resolution, and multimodal 3D anatomical reconstruction of the feline spinal cord, integrating key neuroanatomical structures. Through detailed morphometric analyses and comparisons with human data, we demonstrated strong anatomical similarities, which are essential for successful translation of clinical applications of electrical spinal cord stimulation. We provided important spatial metrics, including dura thickness and circumference, CSF thickness, rootlet distribution and position of the gray matter relative to white matter, that directly impact the design and optimization of both conventional and emerging neuromodulation strategies. By making an anatomically precise 3D model openly available and compatible with finite element platforms like Sim4Life, we allow for reproducible, simulation-ready research that bridges experimental anatomy and computational modeling.

## Data Availability

The datasets presented in this study can be found in online repositories. The names of the repository/repositories and accession number(s) can be found at: https://doi.org/10.5281/zenodo.15333382.
